# Energetic Contributions to Channel Gating of Residues in the Muscle Nicotinic Receptor β1 Subunit

**DOI:** 10.1371/journal.pone.0078539

**Published:** 2013-10-23

**Authors:** Gustav Akk, Megan Eaton, Ping Li, Steven Zheng, Joshua Lo, Joe Henry Steinbach

**Affiliations:** Department of Anesthesiology and the Taylor Family Institute for Innovative Psychiatric Research, Washington University School of Medicine, Saint Louis, Missouri, United States of America; Weizmann Institute of Science, Israel

## Abstract

In the pentameric ligand-gated ion channel family, transmitter binds in the extracellular domain and conformational changes result in channel opening in the transmembrane domain. In the muscle nicotinic receptor and other heteromeric members of the family one subunit does not contribute to the canonical agonist binding site for transmitter. A fundamental question is whether conformational changes occur in this subunit. We used records of single channel activity and rate-equilibrium free energy relationships to examine the β1 (non-ACh-binding) subunit of the muscle nicotinic receptor. Mutations to residues in the extracellular domain have minimal effects on the gating equilibrium constant. Positions in the channel lining (M2 transmembrane) domain contribute strongly and relatively late during gating. Positions thought to be important in other subunits in coupling the transmitter-binding to the channel domains have minimal effects on gating. We conclude that the conformational changes involved in channel gating propagate from the binding-site to the channel in the ACh-binding subunits and subsequently spread to the non-binding subunit.

## Introduction

 The pentameric ligand-gated ion channel (pLGIC) family includes the vertebrate nicotinic, GABA_A_, serotonin-type A and glycine receptors, as well as prokaryotic and invertebrate receptors [1–3]. Each receptor comprises a pentamer of related subunits; the transmitter-binding sites are located at the interface between 2 subunits. The canonical acetylcholine (ACh) binding sites occur between a subunit that contributes the "principal" side and a second subunit that contributes the "complementary" side. In the case of heteromeric pLGIC receptors the result is that 2 pairs of the subunits (4 subunits) contribute to such a transmitter binding site whereas the 5th subunit does not. However, it is clear that the "non-binding subunit" can have profound effects on the activation of the receptor by transmitter [4,5]. Mutations to residues in the channel-lining region of the non-binding subunit affect gating with energetic contributions approximately equal to the effects of homologous mutations in transmitter-binding subunits [6,7]. To date, few studies have been made of the effects on receptor activation of mutations to residues outside the channel-lining region in the non-binding subunit. In the muscle nicotinic receptor the canonical ACh-binding sites are located between the α1 subunit (principal face) and the δ and ε subunits (complementary face). We examined the effects of mutations in the β1 subunit of the muscle nicotinic receptor (that does not bind acetylcholine) to determine the energetic consequences and, when possible, the inferred timing of energetic contributions to gating [8–10]. Our results indicate that the amino-terminal extracellular region of the β1 subunit and the regions proposed to be involved in coupling between extracellular and transmembrane domains have few residues that make significant energetic contributions to the overall receptor gating equilibrium. In contrast, residues in the channel-lining region of the β1 subunit do make energetic contributions and the timing indicates that the change occurs later in the gating process than for homologous residues in the α1 subunit. These findings indicate that the transduction of binding energy to gating flows from the binding regions of the transmitter-binding subunits to the channel and only subsequently is transmitted to the non-binding subunit.

## Methods

### Constructs and expression

 Mouse muscle nicotinic subunits (α1, β1, δ, ε) were expressed in HEK293 cells, using the pcDNA3 vector (Invitrogen, San Diego, CA). HEK293 cells were obtained from ATTC (Manassas VA). Mutations were introduced by QuikChange (Stratagene, San Diego, CA) mutagenesis, and the entire subunit was sequenced to verify that no additional mutations were introduced. Cells were transfected using the calcium-phosphate precipitation method [11]. The aligned sequences for the mouse α1, β1, δ and ε subunits are shown in Figure S1, with positions studied indicated.

### Physiological recordings

 One to 3 days after transfection recordings were made in the cell-attached mode, and single channel events were recorded and analyzed [12]. Cells were bathed in recording bath solution (140 mM NaCl, 5 mM KCl, 1 mM MgCl_2_, 2 mM CaCl_2_, 10 mM glucose, and 10 mM HEPES, pH 7.4) .The pipette solution contained (in mM): 142 KCl, 1.8 CaCl_2_, 1.7 MgCl_2_, 5.4 NaCl, and 10 HEPES, pH 7.4 with added choline. Recordings were made at a membrane potential of -50 mV (determined assuming that the reversal potential is at 0 mV) and room temperature (20-24 °C) using an Axopatch 200B amplifier (Molecular Devices, Union City, CA). Signals were low-pass filtered at 10 kHz, digitized with a Digidata 1320 series interface at 50 kHz using pClamp software (Molecular Devices) and analyzed using the QuB Suite (http://www.qub.buffalo.edu). Events were idealized using the SKM routine in QuB. 

 Choline was used as agonist in all cases. We used choline because the apparent channel opening rate constant is low. Recordings were made at a low concentration (50-100 μM) to determine the apparent channel closing rate constant (k_c_). The apparent opening rate (k_o_) was determined from recordings using a high concentration (20 mM) of choline. Because k_o_ is low, at low concentration apparent openings contain only a single opening, so the apparent closing rate (k_c_) can be readily measured. At high concentration the closed periods reflecting channel opening are clearly resolvable in the experimental data. For wild-type receptors and 9 mutations k_o_ was estimated at both 10 mM and 20 mM choline, to verify that the k_o_ was estimated at a saturating concentration. In no case did the estimates differ significantly between the two concentrations (data not shown), so it is likely that our estimates do not significantly underestimate the actual k_o_.

 We adopted the methods introduced by the Auerbach Laboratory [9] to examine the consequences of mutations on channel gating. The di-liganded equilibrium gating constant (E_2_) was computed as the ratio k_o_ / k_c_ for each mutation at a position. The "range energy" for that position was estimated from the range of values for E_2_ (including wild-type) as 0.59 (ln(E_2,max_ )-ln(E_2,min_)) kcal/mol [8]. The range energy is defined only on the basis of the tested mutations, and so is a minimal estimate of the possible value. The obtained values for k_o_, k_c_ and E_2_ are shown in Table S3. The mutations made had a range of divergence from the original residue, as assessed from the BLOSUM62 [13] value, but the estimated range energy did not depend on the most negative BLOSUM62 score at a location (Figure S3). 

 The parameter φ was calculated from the slope of a logarithmic plot of k_o_ on E_2_ (a "rate-equilibrium plot"). The value of φ has been interpreted to reflect the position along the reaction coordinate from closed to open at which a residue has an effect on the overall energetic change in channel opening. A value near 1 indicates that at the transition state between closed and open the environment at that position is similar to the open state, and a value near 0 that the environment is closed-like [10,14,15]. Rate-equilibrium plots are shown for all the positions examined in Figure S2.

### Molecular model

 The homology model for the mouse muscle receptor was made by threading the mouse sequences (α1: NP_031415.2, β1: NP_033731.3, δ: NP_067611.2, ε: NP_033733.1) on the crystal structure of the glutamate-activated chloride channel from *C. elegans* (GluCl; 3RHW; [16]) using the SWISS-MODEL web tool (http://swissmodel.expasy.org/). Structures were visualized and displays generated using Chimera 1.6.2 (http://www.cgl.ucsf.edu/chimera). The GluCl structure was chosen as the template based on reports [17–19] that it is a better representation of the muscle receptor structure than the *Torpedo marmorata* cryoelectron microscopic structure [20]. The positions of the transmembrane regions differ in the two structures (see Figure S1). We have chosen to call residues that are in the second transmembrane region (TM2) in both structures "TM2," while those that are placed in TM2, TM3 or the linker between them in the different structures as "TM2-link-TM3." 

## Results

**Figure 1:**
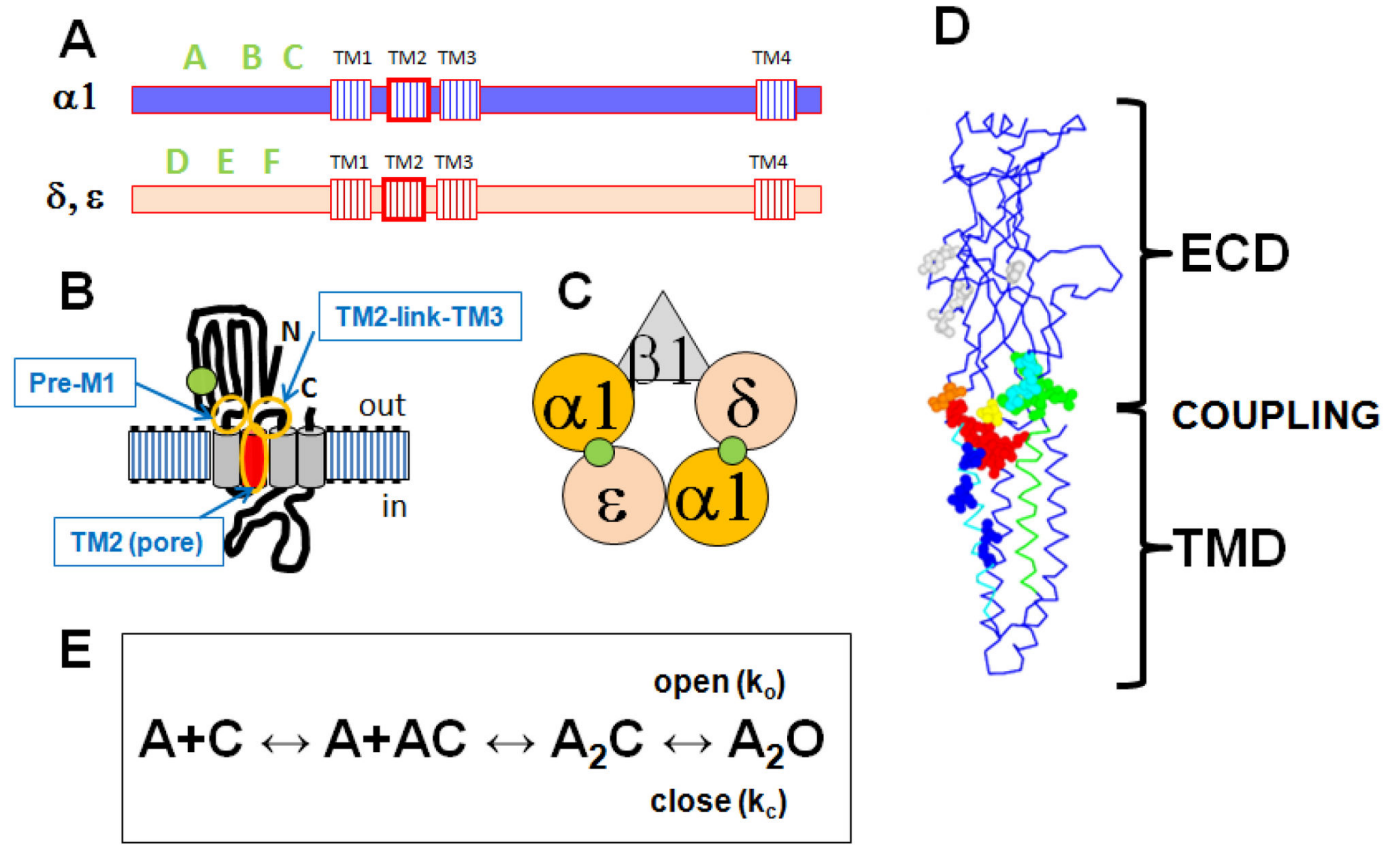
Schematic summaries of AChR structure. **Panel** **A** shows the locations of relevant regions in the primary sequences. There are 4 transmembrane regions (TM1-TM4). The channel is formed by the TM2 regions (highlighted in red) from the 5 subunits. The ACh-binding loops are in the N-terminal extracellular region (loops A-C form the "principal" side, D-F the "complementary" side). **Panel** **B** shows then membrane topology of a subunit (ACh-binding site: green circle, TM2 helix red cylinder). **Panel** **C** shows the arrangement of subunits in the pentamer (viewed from the extracellular side; green circles - ACh-binding interfaces). The channel is located in the center of the rosette of subunits. **Panel** **D** shows a homology model of the mouse β1 subunit, threaded on the *C. elegans* GluCl E subunit [16]. The main chain is shown in blue, and the extracellular domain (ECD) and transmembrane domain (TMD) are indicated by brackets. The region coupling the ECD to the TMD is indicated by "coupling," and includes residues in the PreM1, loop 9, the TM2-link-TM3 region and the "principal pathway" (see text). In the ECD mutated residues in "loop 9" are shown in cyan, "Pre M1" in green, K46 in orange, V132 in yellow and other residues in light gray. In the TMD mutated residues in "TM2" are in blue and in the TM2-link-TM3 region in red. Many of the residues studied are located at or near the interface between the ECD and TMD regions of the subunit. Panel E shows the kinetic scheme used to interpret the data. A receptor with a closed channel (C) binds 2 molecules of agonist (A) then the channel opens (O) with an apparent opening rate k_o_ and apparent closing rate k_c_.

 The adult muscle nicotinic receptor (AChR) is a pentamer containing 2 copies of the α1 subunit and 1 each of the β1, δ and ε subunits (Figure 1). The 2 canonical binding sites for acetylcholine (ACh) and other cholinergic agonists are located at the interfaces between the α1 and δ subunits and the α1 and ε subunits. The α1 subunits contribute the "principal" side of the binding site, with regions named the A, B and C loops. The δ or ε subunits contribute the complementary side, with regions named the D, E and F loops. The β1 subunit does not contribute to a canonical binding site. After binding of transmitter, a series of conformational changes takes place between the transmitter-binding site (located in the extracellular portion of the receptor) and the channel (located in the membrane-spanning portion) that results in channel opening. Several regions of the α1 subunit have been proposed to be essential for establishing the connection between the extracellular domain (ECD) and the transmembrane domain (TMD), including the "PreM1" and "TM2-link-TM3" regions [21,22]. 

 We wished to obtain a picture of the β1 subunit in terms of its energetic contributions to overall channel gating, to compare to those of subunits that bind transmitter. The β1 subunit has not been extensively studied: at least 113 positions have been studied in the α1 subunit, 52 in the ε, 29 in δ but only 15 in β1 (see references to Table S1). We examined positions in the β1 subunit that are homologous to positions that had already been studied in other subunits of the AChR. We made mutations to 27 positions, 13 in the ECD and 14 in transmembrane regions (summarized in Table 1; sequences shown in Figure S1). In general, we chose positions for which relatively large energetic changes had been reported for one or more subunits. In the ECD some positions corresponding to ACh-binding loops in other subunits were studied. We also examined positions proposed to be involved in coupling binding to gating in the α1 subunit. In the TMD we extended the analysis of residues in the transmembrane regions. 

**Table 1:** Mutations created and analyzed.

Residue	Structure	mutations	φ ± SE	Range energy (kcal/mol)
K46	PP	Q, M, R	0.29 ± 0.58	0.43
Y55	D loop	F, Q, S	0.56 ± 0.38	0.24
L93	A loop	A, T, Y	0.52 ± 0.32	0.47
N96	Col	S, V*, W	0.07 ± 0.24	0.52
**V132**		A, Q, L	**-0.12 ± 0.10**	**0.77**
Y149	B loop & Col	A, D, Q, S	0.73 ± 0.26	0.48
N182		D, E, S	0.15 ± 0.29	0.18
G183		F, W, Y	0.69 ± 0.15	0.55
Q184		T, W	-0.37 ± 0.46	0.40
I218	Pre-M1	T, V	0.69 ± 0.66	0.25
**R219**	Pre-M1	I, K, Q	**0.27 ± 0.07**	**0.76**
R220	Pre-M1 & PP	I*, Q, K	6.88 ± 7.88	0.02
K221	Pre-M1	I, Q, R	0.52 ± 0.28	0.54
S257	TM2	C, G, I	-0.05 ± 0.32	0.67
**A260**	TM2	C, G, V	**0.03 ± 0.25**	**0.80**
**T265**	TM2	P, S, Y	**0.42 ± 0.31**	**0.93**
**V266**	TM2 & Col	A, F, T	**0.48 ± 0.07**	**2.01**
**L270**	TM2	A, T, Y	**0.53 ± 0.18**	**2.80**
**V275**	TM2-link-TM3	A, L, M	**0.24 ± 0.27**	**1.19**
**P276**	TM2-link-TM3 & Col	G, K, T	**0.24 ± 0.27**	**1.26**
L280	TM2-link-TM3 & PP	A, T, Y	-0.30 ± 0.27	0.50
A281	TM2-link-TM3	F, T, W	0.34 ± 0.24	0.45
V282	TM2-link-TM3	A, Q, L	0.31 ± 0.08	0.67
P283	TM2-link-TM3 & PP & Col	A, G, S	0.16 ± 0.28	0.62
**I284**	TM2-link-TM3	F, L, T	**0.52 ± 0.12**	**0.78**
**I285**	TM2-link-TM3	F, L, T	**0.24 ± 0.15**	**1.44**
**I286**	TM2-link-TM3	F, L, T	**0.48 ± 0.04**	**1.03**

The first column shows the residue studied. The second shows structural features, where PP indicates a residue in the Principal Pathway and "Col" a residue in the high energy column (see Text). The 3rd column shows the mutations made (an asterisk indicates that no functional channels were recorded from receptors containing that mutated subunit). The 4th column gives φ ± the estimated SE returned by the fitting program. The final column gives range energy (kcal/mol). Positions at which the range energy is greater than 0.7 kcal/mol are shown in **bold**.

**Figure 2:**
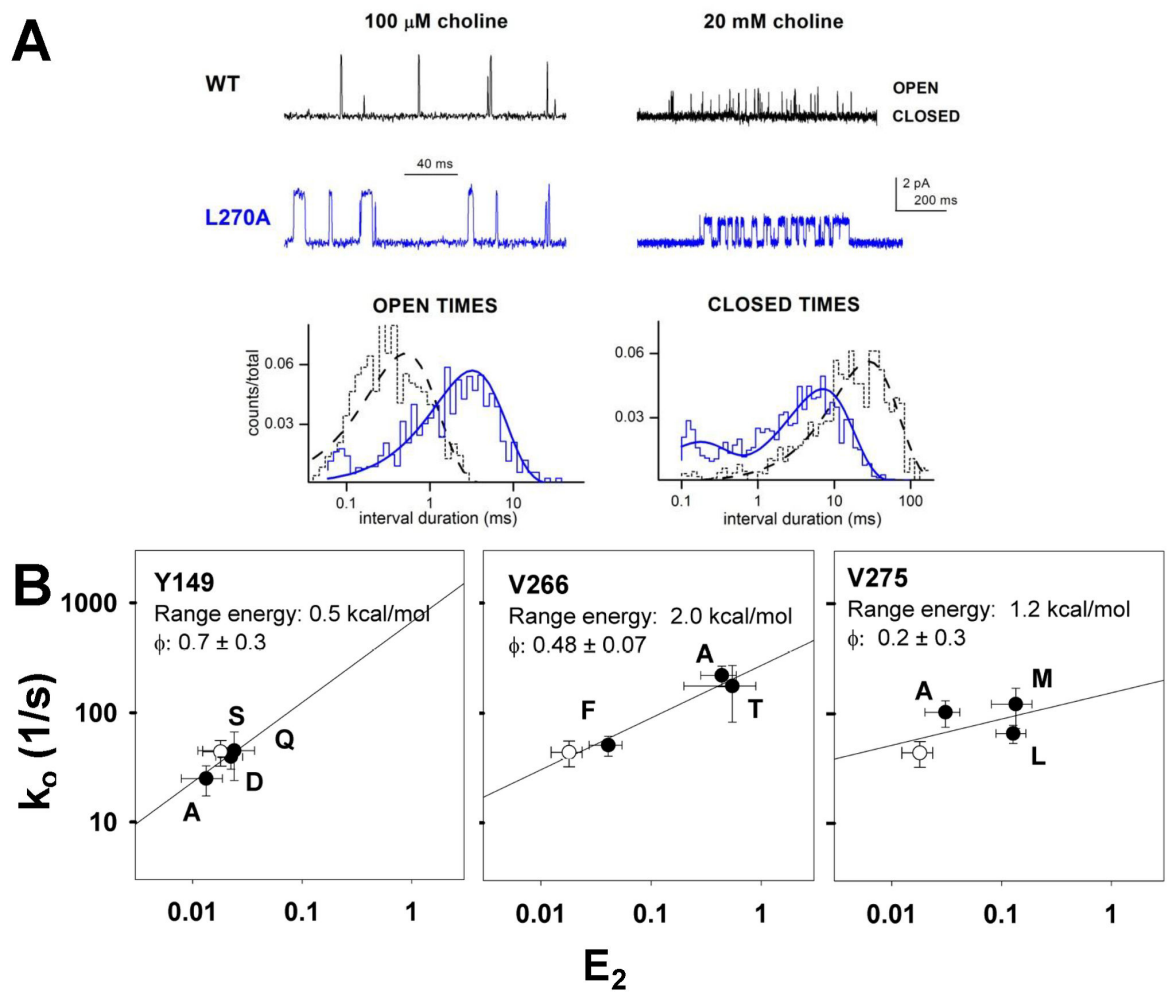
Rate equilibrium free energy relationships. **Panel** **A** shows sample traces of data for wild-type receptors and receptors containing β1(L270A). On the left data at a low concentration (100 μM) of choline are shown, with histograms of the open durations shown below. The fits to the histograms were used to estimate the channel closing rate. The openings are clearly prolonged by the mutation, and the estimates for k_c_ are 2128 s^-1^ for wild type and 360 s^-1^ for the mutant. The number of events in the histogram are 506 for β1(L270A) and 695 for wild type. On the right data at a high concentration (20 mM) are shown, with histograms of the closed durations below. The estimates for k_o_ are 44 s^-1^ for wild type and 153 s^-1^ for the mutant (estimated from the major, slower component). The number of events in the histogram are 2390 for β1(L270A) and 2259 for wild type. Note that open channel block reduces the channel current amplitude at this high concentration. **Panel** **B** shows logarithmic plots of k_o_ on E_2_ for mutations at 3 positions in the β1 subunit. The lines show the linear regression of log(k_o_) on log(E_2_). The range energy is calculated from the range of E_2_ values and φ is the slope of the linear regression (given as regression value ± SE of fit value). β1(Y149) illustrates a position at which the range energy is small, β1(V266) a position with a linear relationship, and β1(V275) a position at which the slope of the line is poorly defined. The hollow symbol shows data for wild-type receptors. Data points are identified with the residue at the position, and are mean ± SE. Rate-equilibrium plots for all positions are shown in Figure S2.

 At each position we made 2 to 4 mutations, and determined the apparent rates for channel opening (k_o_) and closing (k_c_) (see Figure 1E for the kinetic model for activation; there is further consideration of kinetic models and rates in the Discussion). We used the partial agonist choline for all studies. Representative data are shown in Figure 2.

### "Range energy"

**Figure 3:**
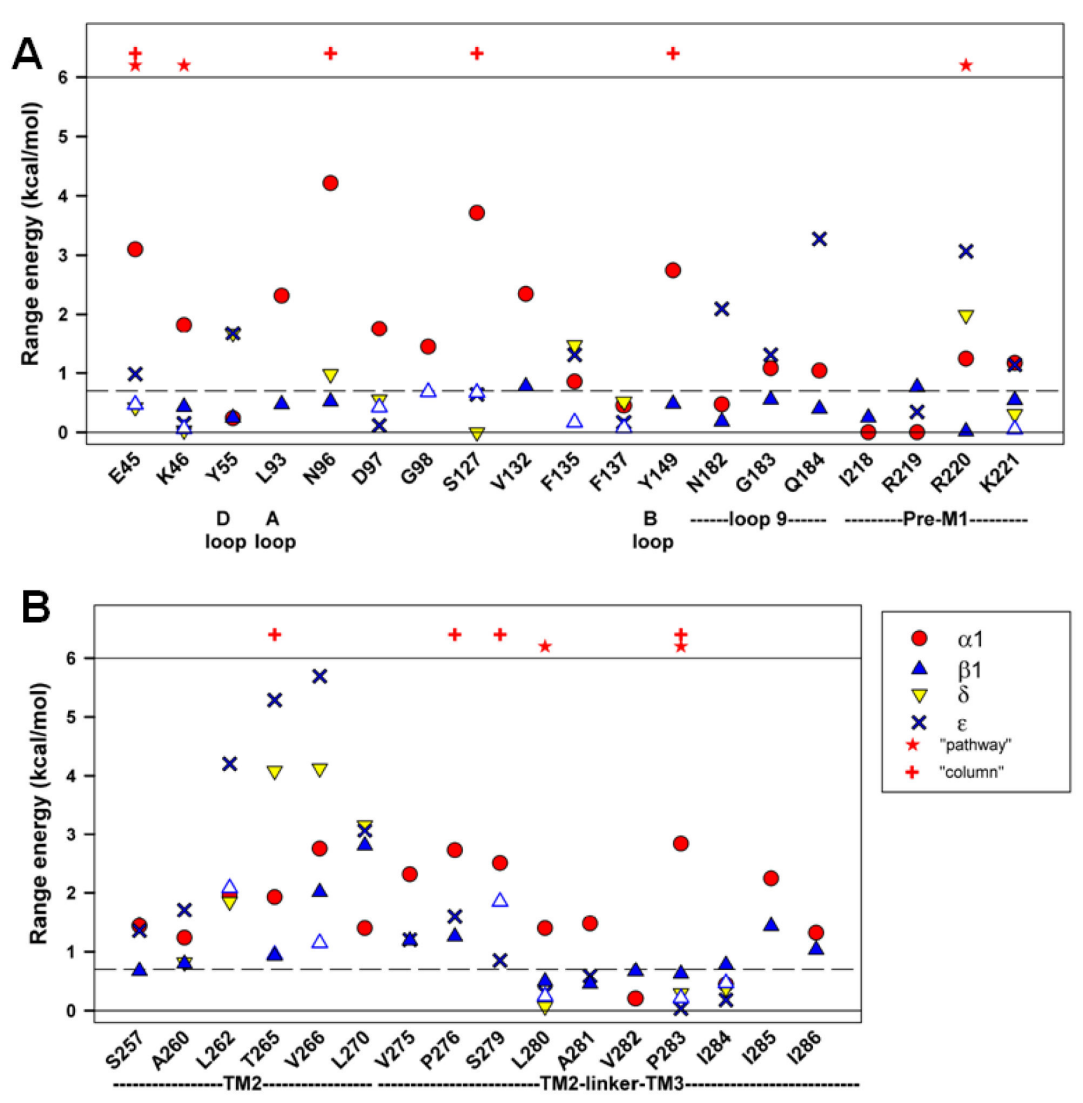
Range energy at homologous positions in the subunits. **Panel** **A** shows data for positions in the ECD, while **panel** **B** shows data for the TMD. The sites are identified by the residue and position in the β1 subunit, and some structural features are shown below (see Text). Residues proposed to be part of the "principal pathway" for coupling binding to gating in the α1 subunit [25] are indicated by stars above the data, and residues lying in a "high range energy column" in the α1 subunit [8] are indicated by plus signs. Results for the β1 subunit are shown by blue triangles (results from the present study shown as filled triangles, while results obtained by others as open triangles). Values for α1 are shown with filled red circles, for δ by yellow inverted triangles and ε by bold crosses. Values are given on a per subunit basis. The dashed line at 0.7 kcal/mol shows the discriminator chosen for the minimal range energy for considering an estimate of φ to be reliable. The values for the present data are shown in Table 1, and all values are shown in Tables S1 and S2.

 We initially characterized the effects of mutations in terms of the ratio of channel opening to closing rate constants (E_2_), to determine the consequences on the overall gating equilibrium. A parameter derived from values of E_2_ is the "range energy" for a given position, defined as the observed maximal range of energetic effects on E_2_. A large value for range energy indicates that the gating equilibrium is highly sensitive to the interactions between the amino-acid residue and its local environment. A small value indicates that that particular position is unlikely to undergo a large change in interactions during opening, at least for the tested substitutions. As can be seen in Figure 3, the range energy is low for positions in the ECD of the β1 subunit, and higher in the TMD. 

 A low value of the range energy might be expected for residues homologous to positions in the transmitter-binding site (the A through F loops), because the β1 subunit does not participate in the canonical ACh-binding site. However, the range energy is also low at other positions in the ECD, including regions proposed to be involved in coupling the extracellular binding domain to the transmembrane channel domain (Table 1, Figures 1 & 3). The locations we studied include some that show high range energies for the δ or ε ACh-binding subunits, including the "loop 9" [23] and "pre-M1" [24] regions. In the β1 subunit, these locations appear to make little contribution to the energetics of gating (Figure 3). Two proposals have been made for residues that are particularly important for coupling binding to gating in the α1 subunit. In one, a complex between the extracellular domain and the transmembrane domain containing α1E45, V46, R209, S269 and P272 was proposed (a “principal pathway” [25]. In the second, a "column" of residues with large range energies was proposed [8], including α1E45, A96, Y127, W149, P265, S268 and P272. Several of these positions were tested in the β1 subunit, and none showed large range energies. We note that previous work has also shown that mutations to some of these positions in non-α subunits have only small effects on gating [25–27]. 

 In the TMD the energetic contributions made by residues in β1 are more comparable to those of other subunits. However, a comparison of our estimates of the range energies for positions in β1 to estimates for homologous positions in other subunits indicates that 11 of 14 positions in β1 have a lower value than for α1. This observation suggests that, overall the energetic contributions from the β1 subunit are lower than those from homologous positions of the α1 subunit. We compared values by simply tabulating the numbers of positions that had lower as opposed to equal or greater values for range energy, and then compared positions in β1 to homologous positions in α1. Based on the binomial distribution with equal probability of being greater or lesser, 11 or more positions would have lower energy with P = 0.03. Similar comparisons of the data from the β1 subunit to data from the δ subunit show 4 of 7 with lower range energy (P = 0.5) and to the ε subunit 8 of 11 have lower range energy (P = 0.11). 

### Timing

 When the range energy is large enough it is possible to accurately calculate the parameter φ, an estimate of the time at which the interaction between a residue and its local environment changes during the channel gating reaction. In practice, φ is calculated as the slope of a plot of the logarithm of k_o_ to the logarithm of E_2_. A value near 1 indicates an early change event, while a value near 0 indicates a later event [10,14,15]. The reliability of the estimate for φ depends on the spread in values for E_2_. To allow for consistency with previously reported values, we adopted the criterion that the range energy needed to be greater than 0.7 kcal/mol (corresponding to a 3.3-fold ratio of values of E_2_) for the estimate to be considered reliable. 

**Figure 4:**
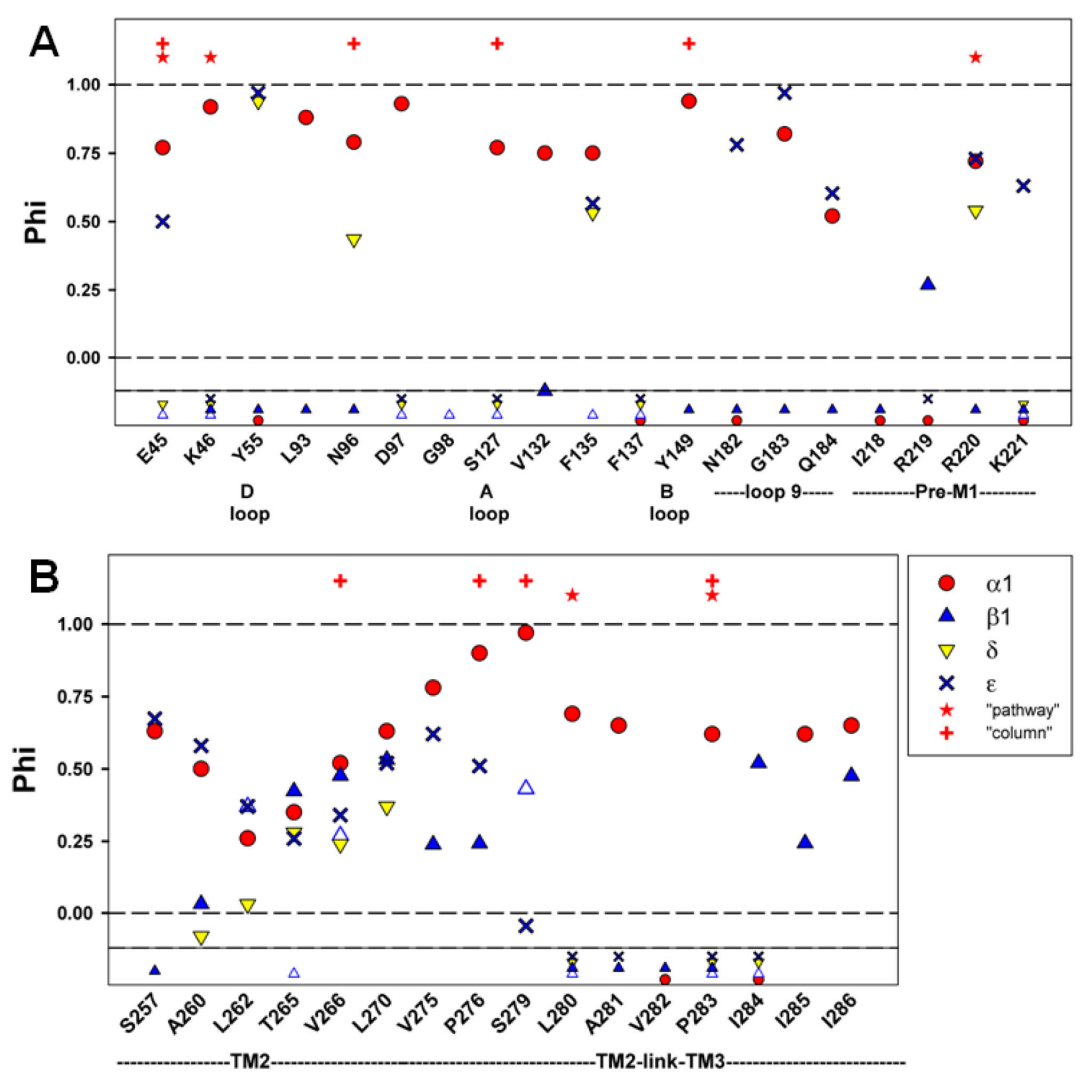
Summary of results for timing. Values are shown for φ, in a similar format to that of Figure 3. The small symbols shown below the solid horizontal line indicate positions at which the range energy was less than 0.7 kcal/mol and for which φ could not be reliably calculated. The values for the present data are shown in Table 1, and all values are shown in Tables S1 and S2.

 Only two residues in the ECD (β1V132 and β1R219) have range energy greater than 0.7 kcal/mol, so little timing information is available in that region (Figure 4; Table 1). Both these residues have lower values for φ than agonist-binding subunits, suggesting late participation.

 In contrast, 10 of 14 residues examined in the TMD had range energy > 0.7 kcal/mol (Figure 3). The comparison of φ values between subunits shows that positions in β1 TMD have lower values (i.e. later timing) in 7 of 8 comparisons to α1 (P = 0.04). However, in comparison to δ no positions in β1 have lower φ (0 of 4, P = 0.06), while in comparison to ε 3 of 6 do (P = 0.5). These comparisons suggest that on the whole the contributions of β1 occur after those of α1, about the same time as those of ε and perhaps before those of δ.

 It is possible that conformational changes at the extracellular end of the channel (that is, the TM2-link-TM3 region) might participate in coupling the ECD and TMD, and so might experience earlier movements during gating. In the α1 subunit residues at this region show larger values for φ than in β1 (4 of 4 comparisons), indicating an earlier movement. Only 2 comparisons could be made to the ε subunit, but in both cases φ for residues in ε was greater. It is interesting to note that the φ values for the β1 subunit are largest and most similar to those of α1 in the middle of TM2 (Figure 4), which might suggest that the conformational change of gating is communicated from the α1 to the β1 subunit at this level.

 As shown in Table 1 several of the residues in the TMD of the β1 subunit have a large uncertainty in our estimate of φ. It has been reported that some positions in other subunits may show non-linear relationships between log(k_o_) and log(E_2_) (for example 28). The reason for this lack of linearity is not known, but the interpretation of φ in terms of timing may be incorrect for these positions. 

### Comparisons across subunits

**Figure 5:**
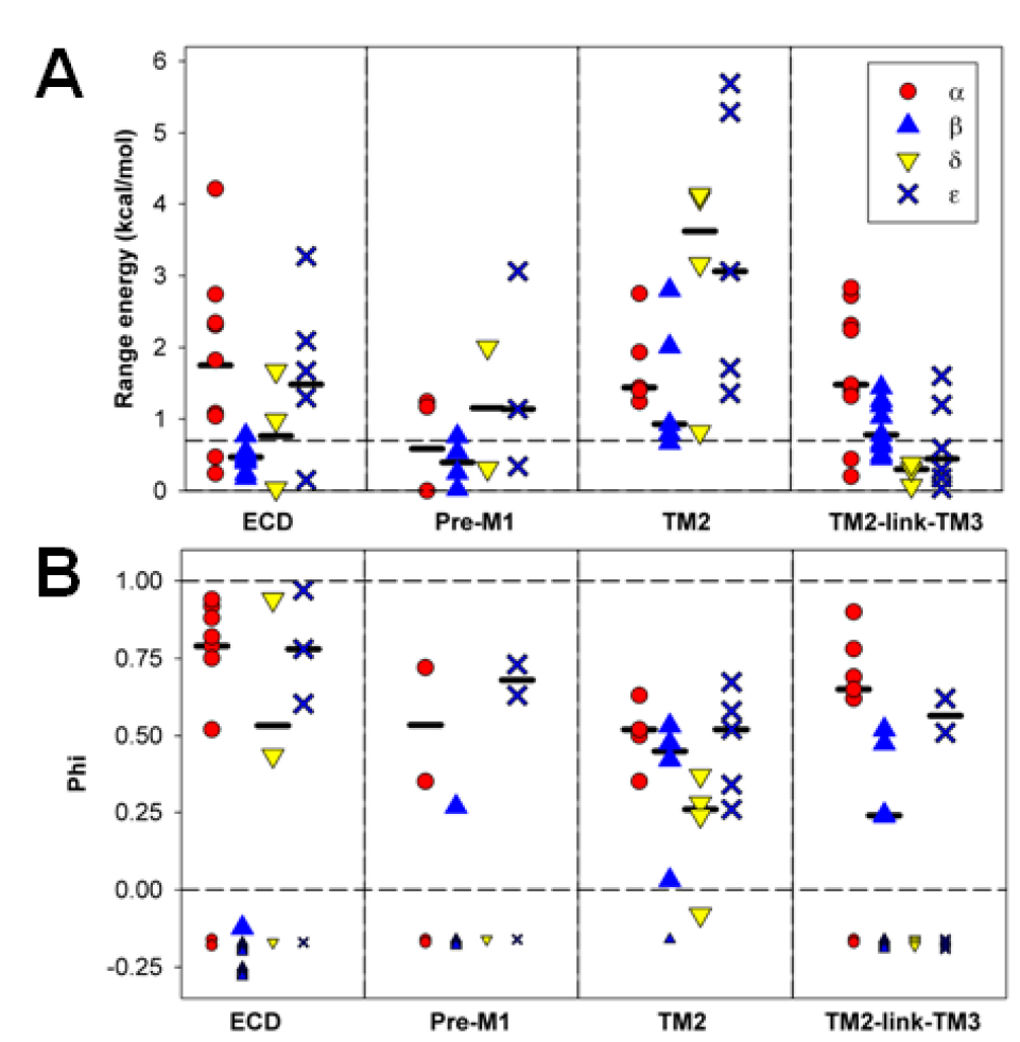
Parameters in subunits divided by structural regions. The data for positions examined in the present study are summarized in terms of the structural region in which the residue is located. The regions shown are ECD (from β1(V46) to Q184), Pre-M1 (β1(I218) to K221), TM2 (β1(S257) to L270) and TM2-link-TM3 (β1(V275) to I286). The upper panel shows data for range energy, with the median values for the individual subunits indicated by the horizontal bars, while the lower panel shows similar data for φ.

 The results obtained are summarized in Figures 3 & 4 for different structural domains in Figure 5. 

**Figure 6:**
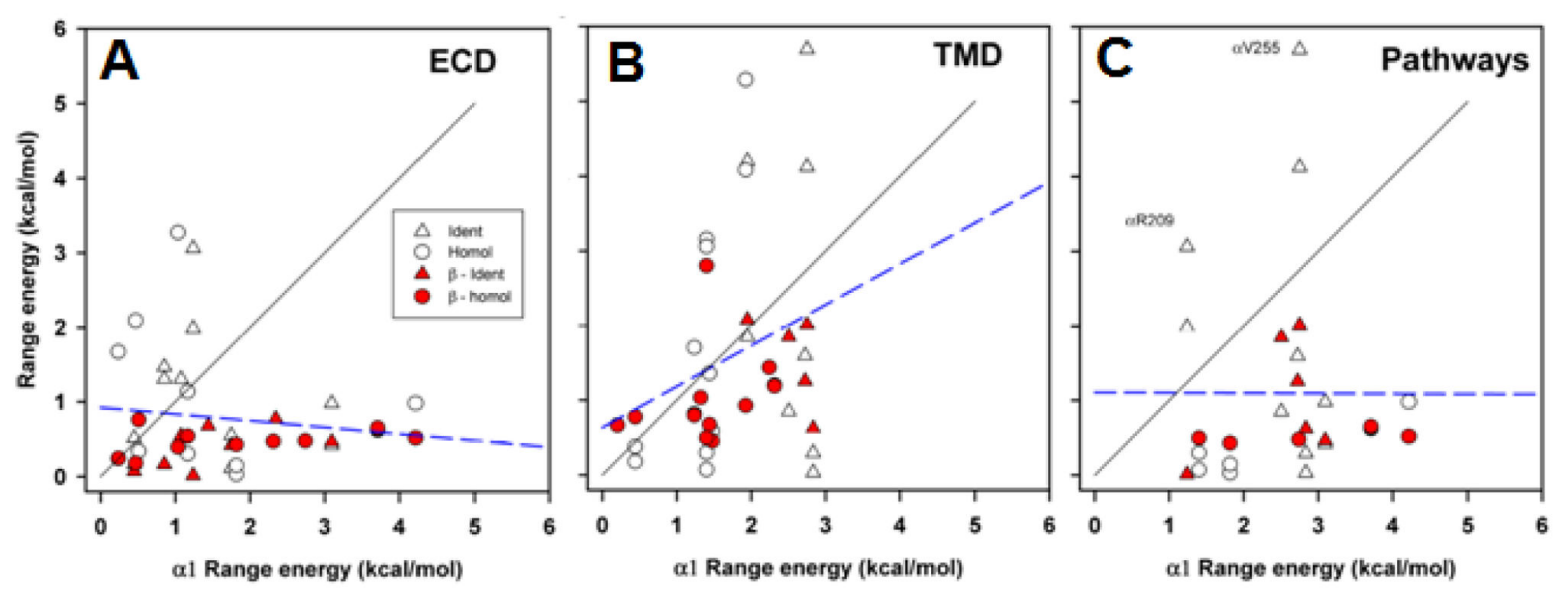
Range energies at homologous positions. Scatter plots for range energy are shown for positions in the β1, δ or ε subunits (ordinate) plotted against the value for the homologous position in α1 (abscissa). All data shown in Figure 3 are plotted. The regions shown are ECD (β1(V46) to K221), TMD (β1(S257) to I286) and Pathways (see Text, β1(E45), K46, N96, S127, Y149, R220, V266, P276, S279, L280 and P283). Two positions are identified by their locations in the α1 subunit, that have large range energies in the complementary face subunits (δ and ε) but lower values in the β1 subunit. Positions at which all 4 subunits have the same amino acid are shown by triangles, while positions at which one or more differ are shown by circles. Data for the β1 subunit are shown as filled red symbols. The solid lines show the line of equality, the dashed lines show the regression lines (no slope differed significantly from 0).

 Previous studies have noted that mutations to homologous positions in different subunits may have very different energetic consequences for channel gating [25]. The present data confirm this observation, as shown in the scatter plots of range energies in Figure 6. The data are presented in terms of the region (ECD in panel A, TMD in panel B) and by whether the residue in α1 is part of the "principal pathway" or high energy column (panel C, "Pathways"). The regression coefficient does not differ significantly from 0 for any comparison (for ECD slope = -0.1, P = 0.39; for TMD slope = 0.55, P = 0.10; for pathway slope = 0.0, P = 0.98). Qualitatively speaking, it seems that positions that have a large range energy in the α1 subunit ECD tend to have low range energy in other subunits, and *vice versa*. In the TMD there is a large scatter of energies but more trend to similar energies in different subunits. This might reflect unique roles of the ECD in the different subunits, but more common roles for homologous residues in the TMD. Overall the proposed activation pathways appear to be unique to the α1 subunit, although both δ and ε show high energetic contributions at the positions homologous to α1R209 ("Pre-M1") and α1V255 (TM2). In contrast, the range energies are lower for β1 at these positions.

## Discussion

 The results indicate that residues in the extracellular domain of the β1 subunit show relatively weak changes in interactions with the local environment during channel gating. The results are not consistent with the idea of circumferential transmission of energy in the extracellular domain; that is, little indication of conformational changes spreading from the transmitter-binding subunits to the β1 subunit through interactions of residues in the ECD. Similarly, residues proposed to be involved in coupling between the ECD and TMD in other subunits do not appear to undergo significant changes in interactions during gating. In contrast, residues in the TMD of the β1 subunit show larger energetic contributions to the gating equilibrium than residues in the ECD. Overall, the results indicate that the β1 subunit makes significant energetic contributions to the gating process only when gating has progressed to the actual channel region. This contribution could reflect conformational changes in the β1 subunit, altered interactions with adjacent subunits, or both.

**Figure 7:**
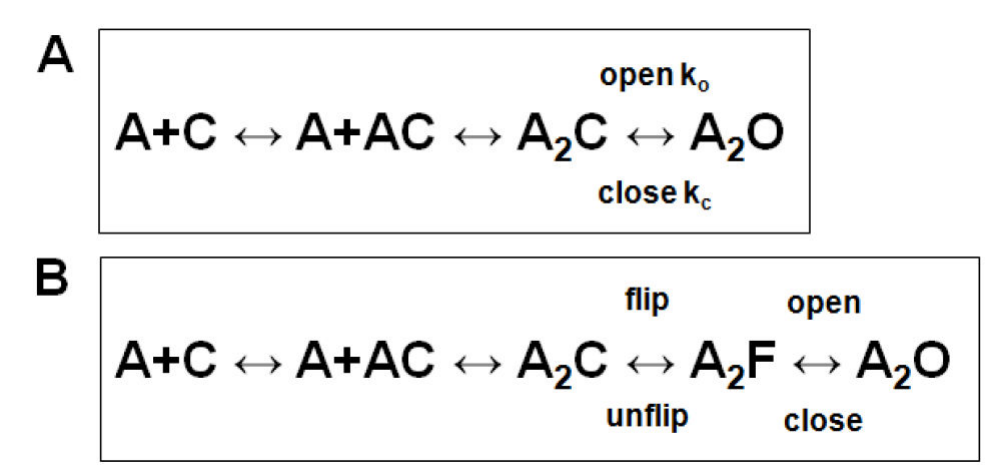
A kinetic model with additional closed states. **Panel** **A** shows the kinetic scheme used in interpreting our data. **Panel** **B** shows an extended scheme incorporating a closed-closed conformational change ("flip") preceeding the channel opening step. In this scheme, A receptor with a closed channel (C) binds 2 molecules of agonist (A) then the receptor enters the flipped state (F) while keeping a closed channel. The channel opens (O) from the flipped state. The scheme is discussed further in the Text.

 We interpreted our observations in terms of a simple kinetic scheme ([29], Figures 1E, 7A) that has been used extensively in studies of the muscle nicotinic receptor . However, a number of recent studies have demonstrated that this model is not adequate, and that there are transitions between closed states that can be kinetically apparent [8,30,31]. A strongly supported version is the "Flip" model, in which there is a closed-closed transition (Flip) immediately before channel opening (Figure 7B) [30]. The data indicate that the actual channel opening rate constant is quite similar between choline and ACh, but that the transition from A_2_C to A_2_F (where F denotes a receptor with a closed channel that is in the "flipped" state) is slower for choline [32]. In the elegant analysis performed by Lape et al. [32] the component in the closed time distribution that we analyzed to obtain an estimate of k_o_ actually reflects dwells of the receptor in all the states preceding A_2_F, and so does not give an estimate of the true channel opening rate constant. This result raises the question of whether the overall interpretation of the observations is appropriate. The goal of our work was to compare the energetic contributions of the β1 subunit to the overall process of receptor activation - the equilibrium between closed channel states and the open state. As such, the kinetic details determining this equilibrium are of lesser importance than the global equilibrium. However, if mutations had different effects when receptor function was probed using different agonists then the comparison might well be between apples and oranges. This seems unlikely to be generally true. In several studies multiple mutations were made a single location, and the effects determined using either choline or ACh as agonist. The data fall along the same line on a logarithmic rate-equilibrium plot, suggesting that the data with the two agonists reflect the same basic energetic consequences of the mutations (for studies with many mutations at a given position see 26,33). However, we note that in two instances mutations do appear to have different consequences for receptors activated by choline than by other agonists: β1(R220Q) (Table S2) and α1(E45R) [34]. We used choline for all constructs tested, and the majority of studies of homologous positions in other subunits used choline for activation of some or all constructs (about 60%, see Table S1). In sum, we feel that our studies of the effects of mutations in the β1 subunit on both the range energy and the timing of energetic contributions can be compared to effects of mutations to homologous residues in other subunits. 

 There were only 2 residues in the ECD of the β1 subunit for which the value of φ could be reliably calculated, but these values indicate late events. In the TMD, for residues at homologous positions α1 appears to have the earliest effects, δ the latest, with β1 and ε intermediate. These observations suggest that the conformational changes involved in gating occur initially in the α1 subunit, then propagate to the β1 and ε subunits and finally to the δ subunit. This difference in timing supports the idea that there is a conformational change in the β1 TMD during channel opening, rather than solely conformational changes in the adjacent subunits that alter the environment around static residues in β1.

 Earlier studies have noted that mutations to homologous residues in the ECD do not have identical effects when made in different subunits [25,35,36]. This may indicate unique roles or dissimilar movements in different subunits. Our observations confirm this observation, and indicate that the ECD in the β1 subunit makes the lowest overall contribution to the gating energy, at least for the locations tested.

 Although this picture shows the β1 subunit in the muscle nicotinic receptor as a relatively passive element in the overall function of the receptor, it is clear that the nature of the subunit that does not contribute to a canonical transmitter-binding site (the "non-binding" subunit) can have a significant effect on the pharmacology and biophysics of a heteropentameric receptor. It can contribute to a binding site for allosteric drugs - most notably for the benzodiazepines acting on the GABA_A_ receptor [37], but also in neuronal nicotinic receptors [38,39] - and in the nicotinic α4β2 receptor there is evidence for a non-canonical ACh-binding site at an α4-α4 interface [39,40]. In addition, the non-binding subunit can alter the gating by transmitters and other agonists [4,5,41]. Similarly, the non-binding subunit can affect the potency of competitive inhibitors [4,42], in which case it is less likely that a change in conformational equilibrium occurs. It has been suggested that the nature of the non-binding subunit may affect the conformation of the pentameric receptor extracellular domain and thereby change the fine structure of the transmitter-binding site [42]. These results suggest a picture in which the non-binding subunit affects the structure of the ECD of the resting receptor. If our observations on the β1 subunit can be generalized to these related receptors, they would generally support this picture with the extension that there is relatively little change in the structure of the ECD for the non-binding subunit during channel gating. However, this then begs the question of the precise intersubunit interactions by which the change in the agonist-binding site is mediated. 

 A previous study examined coupling between the ECD and TMD in a homopentameric receptor, utilizing mutated chimeric subunits formed from the ECD of the nicotinic α7 subunit and the TMD and intracellular domain of the 5HT3_A_ subunit [43]. The "coupling region" studied was a 7 amino acid sequence at the start (extracellular end) of the first transmembrane domain; when these residues were from the α7 subunit channel opening did not occur, while when they were from the 5HT3_A_ subunit it did [44]. The data indicated that the coupling region of a subunit mutated so that it did not contribute to an agonist-binding site did contribute to the overall stability of the open-channel state. This observation is not consistent with our finding that there is no indication that coupling between the ECD and TMD in the β1 subunit affects channel gating of the muscle nicotinic receptor. There are, however, some significant differences between the experiments. First, the studies conducted by Anderson et al [43] were conducted with a basically homopentameric receptor, rather than a heteropentameric receptor. It is possible that the symmetry of the subunits alters some of the structural changes in gating. The second difference is that 6 of the 7 residues swapped in the TM1 region were altered simultaneously. It is possible that this larger perturbation affected gating more generally, rather than coupling specifically. In our experiments we did not mutate any of the residues altered by Anderson et al.

 There are two major caveats to the interpretation of our observations. The first is the possibility that we examined "incorrect" residues in the ECD. Perhaps there is a unique, as yet unidentified, constellation of positions in the β1 subunit which does make a significant contribution to channel gating. The second caveat is the possibility that a change in the β1 subunit takes place as a rigid body motion that may remain undetected by our studies, as they likely focus largely on side-chain interactions. A recent study by Unwin and Fujiyoshi [45], examined cryo-electron microscopic images of AChR from *Torpedo marmorata* that were obtained from specimens frozen within milliseconds after exposure to agonist and were interpreted to reflect the open channel state. The images were compared to previously obtained images, interpreted to reflect the closed channel state [20]. The major change seen in a non-α subunit was a tilt in the β1 ECD (an outward movement of about 0.1 nm). It was proposed that the ECD rocks on the TMD at the interface between the two domains and that this motion is central to the mechanism by which binding is communicated to the gate, indicating a major role of the β1 subunit in channel gating. We mutated many residues at this interface in the β1 subunit (Figure 1), and our data do not indicate a major energetic contribution. Furthermore, the data about timing of energetic contributions in the TMD are not consistent with the idea that conformational changes occur first in the β1 subunit. However, one concern about our data is that it might not have detected changes in position of the backbone polypeptide chain, for example if a tilt "pulled" on the top of TM1 with little change in side-chain interactions. 

 Overall, our results indicate that the β1 subunit is passive in the transfer of the energy of binding from the ECD to the channel gate. However, our data indicate that the non-binding subunit participates in the conformational changes in the channel during opening, and results of others suggest that it may influence the conformation of the transmitter-binding site of the resting receptor. 

## Supporting Information

Table S1 **Data for homologous positions in all subunits.**
(DOCX)

Table S2 **Comparative values for positions in β1.**
(DOCX)

Table S3 **Measured rates.**
(DOCX)

Figure S1 **Aligned primary sequences of the subunits.**
(DOCX)

Figure S2 **REFER plots for the 27 locations.**
(DOCX)

Figure S3 **Range energy versus BLOSUM62.**
(DOCX)

File S1 **The file "Mouse beta1 only on GluCl E Fig 1D.py" is a Python file for Chimera 1.6.2 (http://www.cgl.ucsf.edu/chimera) that contains the homology model shown in Figure 1.**
(PY) Click here for additional data file.
