# Supplementary material for: Energetic Contributions to Channel Gating of Residues in the Muscle Nicotinic Receptor β1 Subunit
Source: PLoS One. 2013 Oct 23;8(10):e78539. doi: 10.1371/journal.pone.0078539 (PMC3806828; doi:10.1371/journal.pone.0078539)
Supplement: Table S1 — Data for homologous positions in all subunits. (DOCX) [file pone.0078539.s001.docx]

Table S1. Data for homologous positions in other subunits.

| Struct | α1 | ϕ | kcal/mol | Ag | Ref | β1 | ϕ | kcal/mol | Ag | Ref | δ | ϕ | kcal/mol | Ag | Ref | ε | ϕ | kcal/mol | Ag | Ref |
| --- | --- | --- | --- | --- | --- | --- | --- | --- | --- | --- | --- | --- | --- | --- | --- | --- | --- | --- | --- | --- |
| PP & Col | ***E45** | **0.77** | **3.09** | both | 16 | E45 | 0.25 | 0.47 | ACh | 13 | E47 | 0.15 | 0.42 | ACh | 13 | **E45** | **0.5** | **0.98** | Cho | 3 |
| PP | **V46** | **0.92** | **1.82** | ACh | 13 | K46 | 0.29 | 0.43 | Cho |  | V48 | 2.59 | 0.03 | ACh | 13 | K46 | -0.8 | 0.15 | ACh | 13 |
| loop D | R55 | 0.66 | 0.24 | Cho | 5 | Y55 | 0.56 | 0.24 | Cho |  | **W57** | **0.94** | **1.67** | ACh | 2 | **W55** | **0.97** | **1.67** | ACh | 2 |
| loop A | **Y93** | **0.88** | **2.31** | ACh | 19 | L93 | 0.52 | 0.47 | Cho |  |  |  |  |  |  |  |  |  |  |  |
| Col | **A96** | **0.79** | **4.21** | both | 4 | N96 | 0.07 | 0.52 | Cho |  | **N98** | **0.44** | **0.98** | Cho | 6 |  |  |  |  |  |
|  | ***D97** | **0.93** | **1.75** | Cho | 6 | D97 | 0.31 | 0.42 | Cho | 6 | D99 | 0.32 | 0.55 | Cho | 6 | D97 | 0.31 | 0.11 | Cho | 6 |
|  | ***G98** | **nd** | **1.44** | Cho | 6 | G98 | 0.4 | 0.68 | Cho | 6 |  |  |  |  |  |  |  |  |  |  |
| Col | **Y127** | **0.77** | **3.71** | both | 18 | S127 | -0.73 | 0.65 | Cho | 18 | S129 | -0.69 | 0.62 | ACh | 18 | T127 | -0.08 | 0.64 | both | 18 |
|  | ***V132** | **0.75** | **2.34** | ACh | 9 | **V132** | **-0.12** | **0.77** | Cho |  |  |  |  |  |  |  |  |  |  |  |
|  | ***F135** | **0.75** | **0.85** | both | 9 | F135 | -0.69 | 0.17 | ACh | 12 | **F137** | **0.53** | **1.47** | ACh | 12 | **F135** | **0.57** | **1.31** | ACh | 12 |
|  | *F137 | 0.53 | 0.45 | Cho | 5 | F137 | -1.34 | 0.07 | ACh | 12 | F139 | 0.39 | 0.52 | ACh | 12 | F137 | 0.96 | 0.17 | ACh | 12 |
| loop B & Col | **W149** | **0.94** | **2.74** | ACh | 15 | Y149 | 0.73 | 0.48 | Cho |  |  |  |  |  |  |  |  |  |  |  |
| loop9 | S173 | 0.14 | 0.47 | both | 10 | N182 | 0.15 | 0.18 | Cho |  |  |  |  |  |  | **N182** | **0.78** | **2.09** | Cho | 10 |
| loop9 | ***G174** | **0.82** | **1.08** | both | 10 | G183 | 0.69 | 0.55 | Cho |  |  |  |  |  |  | **G183** | **0.97** | **1.30** | Cho | 10 |
| loop9 | **E175** | **0.52** | **1.04** | ACh | 10 | Q184 | -0.37 | 0.40 | Cho |  |  |  |  |  |  | **E184** | **0.60** | **3.27** | Cho | 10 |
| Pre-M1 | M207 | 0.05 | 0.24 | Cho | 16 | I218 | 0.69 | 0.25 | Cho |  |  |  |  |  |  |  |  |  |  |  |
| Pre-M1 | Q208 | 0.64 | 0.51 | Cho | 16 | **R219** | **0.27** | **0.76** | Cho |  |  |  |  |  |  | R217 | 0.59 | 0.34 | Cho | 3 |
| Pre-M1 & PP | ***R209** | **0.72** | **1.24** | both | 16 | R220 | 6.88 | 0.02 | Cho |  | **R223** | **0.54** | **1.99** | ACh | 3 | **R218** | **0.73** | **3.06** | ACh | 3 |
| Pre-M1 | **L210** | **0.35** | **1.17** | Cho | 16 | K221 | 0.52 | 0.54 | Cho |  | K224 | 0.82 | 0.31 | ACh | 12 | **K219** | **0.63** | **1.14** | Cho | 3 |
| TM2 | **S246** | **0.63** | **1.44** | Cho | 17 | S257 | -0.05 | 0.67 | Cho |  |  |  |  |  |  | **S256** | **0.67** | **1.36** | ACh | 11 |
| TM2 | **V249** | **0.50** | **1.24** | Cho | 17 | **A260** | **0.03** | **0.80** | Cho |  | **V263** | **-0.08** | **0.82** | Cho | 7 | **V259** | **0.58** | **1.71** | both | 11 |
| TM2 | ***L251** | **0.26** | **1.95** | both | 17 | **L262** | **0.37** | **2.08** | Cho | 14 | **L265** | **0.03** | **1.85** | Cho | 7 | **L261** | **0.37** | **4.20** | Cho | 11 |
| TM2 | **T254** | **0.35** | **1.93** | Cho | 17 | **T265** | **0.42** | **0.93** | Cho |  | **S268** | **0.28** | **4.08** | Cho | 7 | **T264** | **0.26** | **5.29** | Cho | 11 |
| TM2 & Col | ***V255** | **0.52** | **2.75** | both | 17 | **V266** | **0.48** | **2.01** | Cho |  | **V269** | **0.24** | **4.13** | Cho | 7 | **V265** | **0.34** | **5.69** | both | 11 |
| TM2 | **V259** | **0.63** | **1.40** | Cho | 14 | **L270** | **0.53** | **2.80** | Cho |  | **L273** | **0.37** | **3.16** | Cho | 7 | **L269** | **0.52** | **3.06** | Cho | 11 |
| TM2-TM3 | **I264** | **0.78** | **2.31** | both | 1 | **V275** | **0.24** | **1.19** | Cho |  |  |  |  |  |  | **I274** | **0.62** | **1.20** | both | 11 |
| TM2-TM3 & Col | ***P265** | **0.90** | **2.72** | both | 1 | **P276** | **0.24** | **1.26** | Cho |  |  |  |  |  |  | **P275** | **0.51** | **1.60** | Cho | 11 |
| TM2-TM3 & Col | ***S268** | **0.97** | **2.50** | both | 1 | **S279** | **0.43** | **1.85** | Cho | 8 |  |  |  |  |  | **S278** | **-0.04** | **0.85** | both | 11 |
| TM2-TM3 & PP | **S269** | **0.69** | **1.40** | Cho | 8 | L280 | -0.3 | 0.50 | Cho |  | M283 | -0.15 | 0.07 | ACh | 13 | L279 | -0.07 | 0.30 | ACh | 13 |
| TM2-TM3 | **A270** | **0.65** | **1.48** | both | 9 | A281 | 0.34 | 0.45 | Cho |  |  |  |  |  |  | S280 | 0.31 | 0.59 | ACh | 11 |
| TM2-TM3 | V271 | -0.76 | 0.20 | both | 9 | V282 | 0.31 | 0.67 | Cho |  |  |  |  |  |  |  |  |  |  |  |
| TM2-TM3 & Col & PP | ***P272** | **0.62** | **2.83** | both | 9 | P283 | 0.16 | 0.62 | Cho |  | P286 | -0.43 | 0.30 | ACh | 13 | P282 | -5.98 | 0.03 | ACh | 13 |
| TM2-TM3 | L273 | 0.54 | 0.44 | ACh | 9 | **I284** | **0.52** | **0.78** | Cho |  | L287 | 0.01 | 0.38 | ACh | 12 | L283 | 0.67 | 0.18 | ACh | 12 |
| TM2-TM3 | **I274** | **0.62** | **2.24** | ACh | 9 | **I285** | **0.24** | **1.44** | Cho |  |  |  |  |  |  |  |  |  |  |  |
| TM2-TM3 | **G275** | **0.65** | **1.32** | ACh | 9 | **I286** | **0.48** | **1.03** | Cho |  |  |  |  |  |  |  |  |  |  |  |

**Table S1. Data for homologous positions in all subunits.**

The first column shows structural elements in the α subunit (see Text). The following columns show data for each subunit. The residue and position (an asterisk indicates the homologous residues are identical in all subunits), the value of ϕ, the range energy and the source for the data (no source listed indicates data from the present study). When the range energy at a position is greater than 0.7 kcal/mol values are in **bold**. In some cases values for range energy and φ were calculated from data provided in the reference. The column headed "Ag" gives the agonist used in the study: ACh: acetylcholine, Cho: choline, both: both agonists used on different constructs. Ref provides the reference in the list below. If no reference is given then the data are from the present study.

**References for Tables S1 & S2.**

1. Bafna, P.A., P.G. Purohit, and A. Auerbach, Gating at the mouth of the acetylcholine receptor channel: energetic consequences of mutations in the alphaM2-cap. PLoS ONE, 2008. 3(6): p. e2515.

2. Bafna, P.A., A. Jha, and A. Auerbach, Aromatic Residues (epsilon)Trp-55 and (delta)Trp-57 and the Activation of Acetylcholine Receptor Channels. J Biol Chem, 2009. 284(13): p. 8582-8.

3. Bruhova, I. and A. Auerbach, Subunit symmetry at the extracellular domain-transmembrane domain interface in acetylcholine receptor channel gating. J Biol Chem, 2010. 285(50): p. 38898-904.

4. Cadugan, D.J. and A. Auerbach, Linking the acetylcholine receptor-channel agonist-binding sites with the gate. Biophys J, 2010. 99(3): p. 798-807.

5. Chakrapani, S., T.D. Bailey, and A. Auerbach, Gating dynamics of the acetylcholine receptor extracellular domain. J Gen Physiol, 2004. 123(4): p. 341-56.

6. Chakrapani, S., T.D. Bailey, and A. Auerbach, The role of loop 5 in acetylcholine receptor channel gating. J Gen Physiol, 2003. 122(5): p. 521-39.

7. Cymes, G.D., C. Grosman, and A. Auerbach, Structure of the transition state of gating in the acetylcholine receptor channel pore: a phi-value analysis. Biochem, 2002. 41(17): p. 5548-55.

8. Grosman, C., M. Zhou, and A. Auerbach, Mapping the conformational wave of acetylcholine receptor channel gating. Nature, 2000. 403(6771): p. 773-6.

9. Jha, A., et al., Acetylcholine receptor gating at extracellular transmembrane domain interface: the Cys-loop and M2-M3 linker. J Gen Physiol, 2007. 130(6): p. 547-58.

10. Jha, A., et al., The energetic consequences of loop 9 gating motions in acetylcholine receptor-channels. J Physiol, 2012. 590(Pt 1): p. 119-29.

11. Jha, A., P. Purohit, and A. Auerbach, Energy and structure of the M2 helix in acetylcholine receptor-channel gating. Biophys J, 2009. 96(10): p. 4075-84.

12. Lee, W.Y., C.R. Free, and S.M. Sine, Binding to gating transduction in nicotinic receptors: Cys-loop energetically couples to pre-M1 and M2-M3 regions. J Neurosci, 2009. 29(10): p. 3189-99.

13. Lee, W.Y. and S.M. Sine, Principal pathway coupling agonist binding to channel gating in nicotinic receptors. Nature, 2005. 438(7065): p. 243-7.

14. Mitra, A., G.D. Cymes, and A. Auerbach, Dynamics of the acetylcholine receptor pore at the gating transition state. Proc Natl Acad Sci U S A, 2005. 102(42): p. 15069-74.

15. Purohit, P. and A. Auerbach, Glycine hinges with opposing actions at the acetylcholine receptor-channel transmitter binding site. Mol Pharmacol, 2011. 79(3): p. 351-9.

16. Purohit, P. and A. Auerbach, Acetylcholine receptor gating at extracellular transmembrane domain interface: the "pre-M1" linker. J Gen Physiol, 2007. 130(6): p. 559-68.

17. Purohit, P., A. Mitra, and A. Auerbach, A stepwise mechanism for acetylcholine receptor channel gating. Nature, 2007. 446(7138): p. 930-3.

18. Purohit, P. and A. Auerbach, Acetylcholine receptor gating: movement in the alpha-subunit extracellular domain. J Gen Physiol, 2007. 130(6): p. 569-79.

19. Purohit, P., I. Bruhova, and A. Auerbach, Sources of energy for gating by neurotransmitters in acetylcholine receptor channels. Proc Natl Acad Sci U S A, 2012. 109(24): p. 9384-9.
