# Supplementary material for: Energetic Contributions to Channel Gating of Residues in the Muscle Nicotinic Receptor β1 Subunit
Source: PLoS One. 2013 Oct 23;8(10):e78539. doi: 10.1371/journal.pone.0078539 (PMC3806828; doi:10.1371/journal.pone.0078539)
Supplement: Table S2 — Comparative values for positions in β1. (DOCX) [file pone.0078539.s002.docx]

**Table S2. Comparative values for positions in β1**

| Res | φ | kcal/mol | N | Ref | φ | kcal/mol | N | Ag | Ref |
| --- | --- | --- | --- | --- | --- | --- | --- | --- | --- |
| K46 | 0.29 | 0.43 | 4 | This study | 2.44 | 0.06 | 2 | ACh | 13 |
| R220 | 6.88 | 0.17 | 3 | This study | **0.44** | **2.30** | 3 | Cho, CCh | 3 |
| K221 | 0.52 | 0.54 | 4 | This study | -0.49 | 0.05 | 2 | ACh | 12 |
| T265 | **0.42** | **0.93** | 4 | This study | **0.66** | **0.95** | 2 | Cho | 8 |
| V266 | **0.48** | **2.01** | 4 | This study | **0.27** | **1.15** | 2 | Cho | 8 |
| L280 | -0.30 | 0.50 | 4 | This study | 0.35 | 0.24 | 2 | ACh | 13 |
| P283 | 0.16 | 0.62 | 4 | This study | 0.15 | 0.21 | 2 | ACh | 13 |
| I284 | **0.52** | **0.78** | 4 | This study | 0.47 | 0.47 | 2 | ACh | 12 |

Eight positions were examined in the present study and previous work. Values are presented as in Table S1, except that N gives the number of constructs examined (including wild-type).

For 6 positions the results are generally similar. At 4 positions (K46, K221, L280, P283) range energies less than 0.7 kcal/mol were found in all studies. At I284 the difference in range energies was also small, although in our study it was larger than 0.7 kcal/mol, and the values for φ were similar. T265 had similar range energies while our φ value was somewhat less. At V266 where both parameters differed; we examined a larger number of mutations, perhaps accounting for the larger range energy.

The eighth position is β1(R220), at which our results differ greatly from published values. We obtained a range energy of 0.17 kcal/mol, while (3) reported 2.30 kcal/mol. Our (unreliable) value for φ is 6.88, as opposed to 0.44. We note that mouse subunits were used in both studies and the same mutations (β1(R220K) and β1(R220Q)) were made. The only difference is that choline (Cho) was used for all receptors in our study, whereas carbamylcholine (CCh) was used for one of the mutations in the previous study. Results for β1(R220K), where choline was used by both studies, are similar whereas the data for β1(R220Q) are divergent (see below). We have confirmed that the mutation β1(R220Q) does have a larger effect on activation by CCh than Cho (see below), although less than previously reported. We have no explanation for the observation. In any case, for this study we have used our observations with a single agonist.

The values for k_o_ and E2 obtained (normalized to data for wild-type receptors) are:

| Res | Agonist | k_o_ | E2 |  |  | Agonist | k_o_ | E2 | Ref |
| --- | --- | --- | --- | --- | --- | --- | --- | --- | --- |
| R220K | Cho | 0.76 | 1.00 | This study |  | Cho | 0.63 | 0.60 | 3 |
| R220Q | Cho | 1.02 | 1.02 | This study |  |  |  |  |  |
| R220Q | CCh | 0.60 | 0.34 | This study |  | CCh | 0.17 | 0.02 | 3 |

**References for Tables S1 & S2.**

1. Bafna, P.A., P.G. Purohit, and A. Auerbach, Gating at the mouth of the acetylcholine receptor channel: energetic consequences of mutations in the alphaM2-cap. PLoS ONE, 2008. 3(6): p. e2515.

2. Bafna, P.A., A. Jha, and A. Auerbach, Aromatic Residues (epsilon)Trp-55 and (delta)Trp-57 and the Activation of Acetylcholine Receptor Channels. J Biol Chem, 2009. 284(13): p. 8582-8.

3. Bruhova, I. and A. Auerbach, Subunit symmetry at the extracellular domain-transmembrane domain interface in acetylcholine receptor channel gating. J Biol Chem, 2010. 285(50): p. 38898-904.

4. Cadugan, D.J. and A. Auerbach, Linking the acetylcholine receptor-channel agonist-binding sites with the gate. Biophys J, 2010. 99(3): p. 798-807.

5. Chakrapani, S., T.D. Bailey, and A. Auerbach, Gating dynamics of the acetylcholine receptor extracellular domain. J Gen Physiol, 2004. 123(4): p. 341-56.

6. Chakrapani, S., T.D. Bailey, and A. Auerbach, The role of loop 5 in acetylcholine receptor channel gating. J Gen Physiol, 2003. 122(5): p. 521-39.

7. Cymes, G.D., C. Grosman, and A. Auerbach, Structure of the transition state of gating in the acetylcholine receptor channel pore: a phi-value analysis. Biochem, 2002. 41(17): p. 5548-55.

8. Grosman, C., M. Zhou, and A. Auerbach, Mapping the conformational wave of acetylcholine receptor channel gating. Nature, 2000. 403(6771): p. 773-6.

9. Jha, A., et al., Acetylcholine receptor gating at extracellular transmembrane domain interface: the Cys-loop and M2-M3 linker. J Gen Physiol, 2007. 130(6): p. 547-58.

10. Jha, A., et al., The energetic consequences of loop 9 gating motions in acetylcholine receptor-channels. J Physiol, 2012. 590(Pt 1): p. 119-29.

11. Jha, A., P. Purohit, and A. Auerbach, Energy and structure of the M2 helix in acetylcholine receptor-channel gating. Biophys J, 2009. 96(10): p. 4075-84.

12. Lee, W.Y., C.R. Free, and S.M. Sine, Binding to gating transduction in nicotinic receptors: Cys-loop energetically couples to pre-M1 and M2-M3 regions. J Neurosci, 2009. 29(10): p. 3189-99.

13. Lee, W.Y. and S.M. Sine, Principal pathway coupling agonist binding to channel gating in nicotinic receptors. Nature, 2005. 438(7065): p. 243-7.

14. Mitra, A., G.D. Cymes, and A. Auerbach, Dynamics of the acetylcholine receptor pore at the gating transition state. Proc Natl Acad Sci U S A, 2005. 102(42): p. 15069-74.

15. Purohit, P. and A. Auerbach, Glycine hinges with opposing actions at the acetylcholine receptor-channel transmitter binding site. Mol Pharmacol, 2011. 79(3): p. 351-9.

16. Purohit, P. and A. Auerbach, Acetylcholine receptor gating at extracellular transmembrane domain interface: the "pre-M1" linker. J Gen Physiol, 2007. 130(6): p. 559-68.

17. Purohit, P., A. Mitra, and A. Auerbach, A stepwise mechanism for acetylcholine receptor channel gating. Nature, 2007. 446(7138): p. 930-3.

18. Purohit, P. and A. Auerbach, Acetylcholine receptor gating: movement in the alpha-subunit extracellular domain. J Gen Physiol, 2007. 130(6): p. 569-79.

19. Purohit, P., I. Bruhova, and A. Auerbach, Sources of energy for gating by neurotransmitters in acetylcholine receptor channels. Proc Natl Acad Sci U S A, 2012. 109(24): p. 9384-9.
