# Supplementary material for: Energetic Contributions to Channel Gating of Residues in the Muscle Nicotinic Receptor β1 Subunit
Source: PLoS One. 2013 Oct 23;8(10):e78539. doi: 10.1371/journal.pone.0078539 (PMC3806828; doi:10.1371/journal.pone.0078539)
Supplement: Table S3 — Measured rates. (DOCX) [file pone.0078539.s003.docx]

Table S3. Measured rates.

| R | Pos | M | k_o_ ± SEM (N) | k_c_ ± SEM (N) | k_o_ / k_c_ |
| --- | --- | --- | --- | --- | --- |
| WT |  |  | 44 ± 5 (6) | 2455 ± 174 (5) | 0.018 |
| K | 46 | M | 23 ± 6 (3) | 2211 ± 300 (3) | 0.011 |
| K | 46 | Q | 22 ± 7 (3) | 1860 ± 248 (3) | 0.012 |
| K | 46 | R | 23 ± 3 (3) | 967 ± 94 (3) | 0.024 |
| Y | 55 | F | 42 ± 6 (4) | 1834 ± 418 (3) | 0.023 |
| Y | 55 | Q | 33 ± 7 (4) | 2024 ± 141 (4) | 0.016 |
| Y | 55 | S | 45 ± 7 (3) | 1858 ± 302 (3) | 0.024 |
| L | 93 | A | 37 ± 5 (5) | 1485 ± 122 (3) | 0.025 |
| L | 93 | T | 66 ± 10 (3) | 1738 ± 93 (3) | 0.038 |
| L | 93 | Y | 57 ± 17 (3) | 1412 ± 180 (4) | 0.040 |
| N | 96 | S | NE | NE | NE |
| N | 96 | V | 44 ± 4 (3) | 1015 ± 110 (3) | 0.043 |
| N | 96 | W | 36 ± 6 (3) | 1621 ± 70 (3) | 0.022 |
| V | 132 | A | 41 ± 7 (3) | 2905 ± 532 (3) | 0.014 |
| V | 132 | L | 37 ± 2 (3) | 712 ± 93 (5) | 0.052 |
| V | 132 | Q | 48 ± 10 (3) | 2562 ± 325 (3) | 0.019 |
| Y | 149 | A | 25 ± 3 (7) | 1869 ± 291 (3) | 0.013 |
| Y | 149 | D | 39 ± 5 (3) | 1757 ± 152 (3) | 0.022 |
| Y | 149 | Q | 48 ± 14 (3) | 1588 ± 177 (4) | 0.030 |
| Y | 149 | S | 45 ± 12 (3) | 1892 ± 233 (4) | 0.024 |
| N | 182 | D | 58 ± 13 (3) | 2406 ± 53 (3) | 0.024 |
| N | 182 | E | 51 ± 6 (3) | 2084 ± 157 (3) | 0.024 |
| N | 182 | S | 52 ± 10 (3) | 3689 ± 292 (3) | 0.014 |
| G | 183 | F | 58 ± 4 (3) | 2947 ± 180 (3) | 0.020 |
| G | 183 | W | 28 ± 3 (4) | 3559 ± 261 (3) | 0.008 |
| G | 183 | Y | 40 ± 1 (3) | 3310 ± 234 (3) | 0.012 |
| Q | 184 | T | 30 ± 4 (4) | 1171 ± 122 (4) | 0.025 |
| Q | 184 | W | 38 ± 5 (3) | 2930 ± 178 (3) | 0.013 |
| I | 218 | T | 28 ± 5 (3) | 2396 ± 266 (3) | 0.012 |
| I | 218 | V | 32 ± (1) | 1793 ± 215 (3) | 0.018 |
| R | 219 | I | 51 ± 12 (3) | 869 ± 29 (3) | 0.058 |
| R | 219 | K | 33 ± 7 (3) | 4660 ± 752 (5) | 0.007 |
| R | 219 | Q | 68 ± 12 (4) | 1041 ± 79 (3) | 0.065 |
| R | 220 | I | NE | NE | NE |
| R | 220 | K | 34 ± 3 (3) | 1879 ± 396 (3) | 0.018 |
| R | 220 | Q | 45 ± 5 (4) | 2440 ± 593 (3) | 0.018 |
| K | 221 | I | 34 ± 5 (3) | 1648 ± 434 (3) | 0.021 |
| K | 221 | Q | 44 ± 5 (4) | 2615 ± 223 (4) | 0.017 |
| K | 221 | R | 66 ± 15 (3) | 1581 ± 334 (4) | 0.042 |
| S | 257 | C | 34 ± 3 (3) | 1088 ± 79 (3) | 0.031 |
| S | 257 | G | 33 ± 2 (4) | 3258 ± 209 (3) | 0.010 |
| S | 257 | I | 26 ± 3 (3) | 1128 ± 91 (3) | 0.023 |
| A | 260 | C | 26 ± 3 (3) | 1382 ± 288 (3) | 0.019 |
| A | 260 | G | 37 ± 7 (3) | 1441 ± 18 (3) | 0.026 |
| A | 260 | V | 36 ± 6 (3) | 514 ± 44 (3) | 0.069 |
| T | 265 | P | 60 ± 2 (2) | 717 ± 64 (4) | 0.083 |
| T | 265 | S | 128 ± 13 (3) | 1473 ± 158 (3) | 0.087 |
| T | 265 | Y | 81 ± 3 (3) | 2002 ± 220 (4) | 0.040 |
| V | 266 | A | 221 ± 27 (3) | 506 ± 84 (3) | 0.437 |
| V | 266 | F | 51 ± 6 (3) | 1257 ± 145 (5) | 0.041 |
| V | 266 | T | 177 ± 54 (3) | 325 ± 66 (3) | 0.545 |
| L | 270 | A | 144 ± 16 (3) | 564 ± 105 (3) | 0.255 |
| L | 270 | T | 79 ± 9 (3) | 235 ± 52 (4) | 0.335 |
| L | 270 | Y | 650 ± 152 (3) | 312 ± 54 (3) | 2.083 |
| V | 275 | A | 103 ± 16 (3) | 3352 ± 331 (5) | 0.031 |
| V | 275 | L | 66 ± 6 (4) | 520 ± 72 (3) | 0.127 |
| V | 275 | M | 122 ± 28 (3) | 909 ± 18 (3) | 0.135 |
| P | 276 | G | 48 ± 13 (3) | 1073 ± 43 (3) | 0.045 |
| P | 276 | K | 28 ± 4 (3) | 674 ± 77 (4) | 0.041 |
| P | 276 | T | 95 ± 19 (3) | 628 ± 17 (4) | 0.151 |
| L | 280 | A | 33 ± 3 (3) | 1187 ± 56 (3) | 0.028 |
| L | 280 | T | 45 ± 6 (3) | 1596 ± 103 (4) | 0.028 |
| L | 280 | Y | 34 ± 4 (3) | 813 ± 231 (3) | 0.042 |
| A | 281 | F | 50 ± 9 (3) | 1286 ± 61 (3) | 0.039 |
| A | 281 | T | 35 ± 4 (3) | 1650 ± 145 (4) | 0.021 |
| A | 281 | W | 51 ± 9 (3) | 1510 ± 110 (3) | 0.034 |
| V | 282 | A | 42 ± 7 (3) | 2387 ± 228 (5) | 0.018 |
| V | 282 | L | 55 ± 6 (3) | 998 ± 123 (4) | 0.055 |
| V | 282 | Q | 31 ± 2 (3) | 3195 ± 332 (4) | 0.010 |
| P | 283 | A | 36 ± 6 (3) | 717 ± 133 (4) | 0.050 |
| P | 283 | G | 63 ± 10 (4) | 1251 ± 58 (3) | 0.050 |
| P | 283 | S | 37 ± 6 (4) | 2106 ± 232 (3) | 0.017 |
| I | 284 | F | 38 ± 9 (3) | 1734 ± 252 (3) | 0.022 |
| I | 284 | L | 61 ± 6 (3) | 1445 ± 79 (3) | 0.042 |
| I | 284 | T | 80 ± 15 (4) | 1193 ± 31 (3) | 0.067 |
| I | 285 | F | 38 ± 7 (3) | 1107 ± 61 (3) | 0.035 |
| I | 285 | L | 33 ± 5 (3) | 782 ± 20 (3) | 0.043 |
| I | 285 | T | 71 ± 11 (3) | 348 ± 75 (3) | 0.205 |
| I | 286 | F | 97 ± 2 (3) | 1177 ± 103 (4) | 0.082 |
| I | 286 | L | 41 ± 6 (4) | 3321 ± 118 (3) | 0.012 |
| I | 286 | T | 41 ± 5 (4) | 2880 ± 490 (3) | 0.014 |

Table S3. Measured rates. The first column identifies the wild-type residue, the second its position in the mature subunit, and the third the mutation made. The next columns give the apparent opening rate (k_o_), apparent closing rate (k_c_) and the ratio of the opening to closing rates. Rates are given in 1/sec, and the number of patches analyzed is N. NE: no channel openings recorded.
