# Supplementary material for: Energetic Contributions to Channel Gating of Residues in the Muscle Nicotinic Receptor β1 Subunit
Source: PLoS One. 2013 Oct 23;8(10):e78539. doi: 10.1371/journal.pone.0078539 (PMC3806828; doi:10.1371/journal.pone.0078539)
Supplement: Figure S1 — Aligned primary sequences of the subunits. (DOCX) [file pone.0078539.s004.docx]

**Figure S1. Aligned primary sequences of the subunits.**

The aligned sequences of the mature subunits from mouse are shown. The signal sequences are not shown, and the sequences are truncated immediately after TM3. Yellow highlighting indicates residues that are identical in all 4 subunits, emphasizing the high degree of similarity. The first line above the sequences shows named structural regions. Regions contributing to the canonical ACh-binding site are shown highlighted in pink (A, B, C in the α1 subunit) or blue (D, E, F in δ or ε). The cysteine loop characteristic of pLGIC ("y") and the "Loop 9" region ("l9") are shown in gray . The "Pre-M1" region is highlighted in orange ("p"). The line above that indicates positions that have been studied in one or more of the α1, δ or ε subunits, marked by an "x". Finally, the top line indicates residues in the β1 subunit that have been studied by downward arrows. Residues examined in the present work have an orange highlight on the arrows. The position of the transmembrane helices differs in the two available structures: they are indicated by green boxes either above the sequences (from cryo-EM images (Unwin, J Mol Biol. **346**:967-89, 2005)) or below the sequences (from the GluCl structure (Hibbs & Gouaux, Nature. 474:54-60, 2011)). Note that the disagreement in position means that the location of the TM2-TM3 linker is unclear. In the text we refer to residues included in TM2 in both structures as in "TM2," while residues that are assigned to TM2, the TM2-TM3 linker or TM3 are referred to as "TM2-link-TM3."
