# Supplementary material for: Energetic Contributions to Channel Gating of Residues in the Muscle Nicotinic Receptor β1 Subunit
Source: PLoS One. 2013 Oct 23;8(10):e78539. doi: 10.1371/journal.pone.0078539 (PMC3806828; doi:10.1371/journal.pone.0078539)

**Figure S2. REFER plots for the 27 locations.**


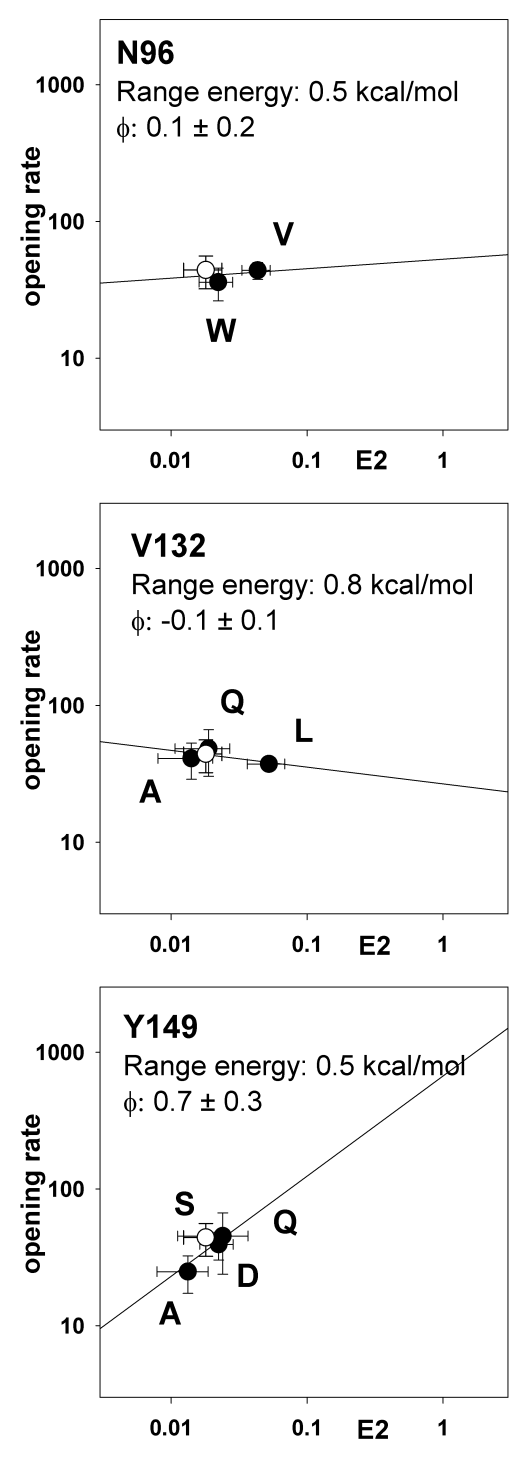


**Figure S2. REFER plots for the 27 locations (continued).**

**Figure S2. REFER plots for the 27 locations (continued).**


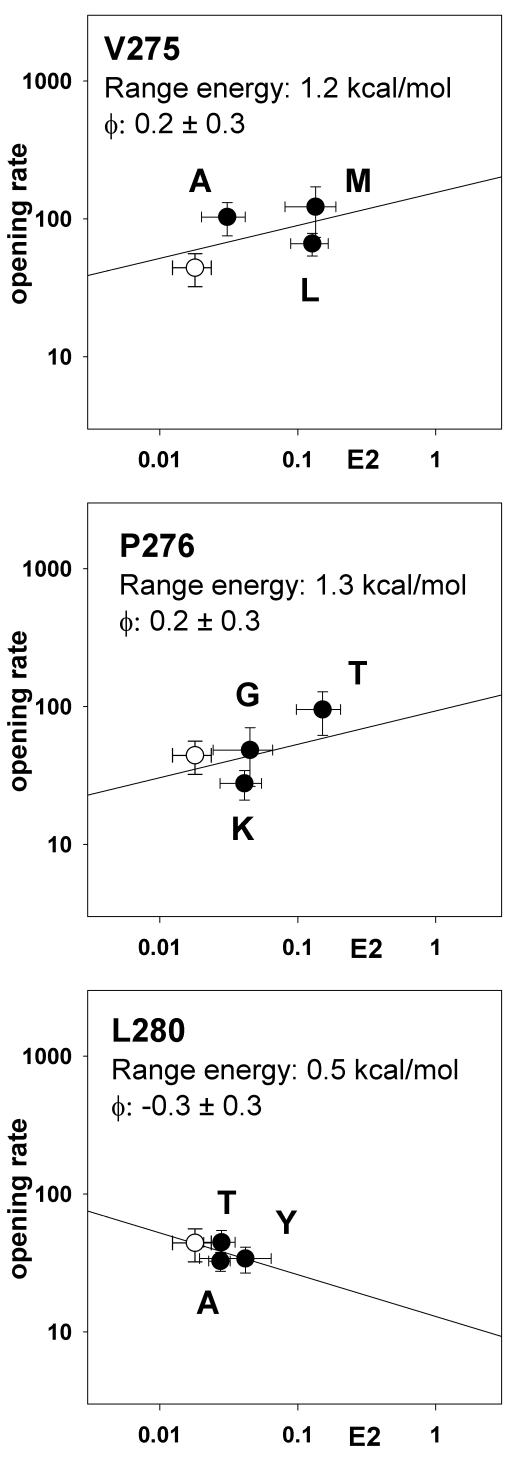

Supplement: Figure S2 — REFER plots for the 27 locations. (DOCX) [file pone.0078539.s005.docx]
