# Supplementary material for: Energetic Contributions to Channel Gating of Residues in the Muscle Nicotinic Receptor β1 Subunit
Source: PLoS One. 2013 Oct 23;8(10):e78539. doi: 10.1371/journal.pone.0078539 (PMC3806828; doi:10.1371/journal.pone.0078539)
Supplement: Figure S3 — Range energy versus BLOSUM62. (DOCX) [file pone.0078539.s006.docx]

**Figure S3. Range energy versus BLOSUM62.**

The range energy (kcal/mol) is plotted against the minimal BLOSUM62 score for the mutations made at a position in the β1 subunit (filled symbols) and the α1, δ and ε subunits (open symbols). The minimal score was chosen to emphasize the consequences for the least likely substitution. The median values (red "plus" and solid line) show a slight trend to lower range energies with higher minimal score, but the regression slope (red dashed line) is not significantly different from 0 (P = 0.4).
